# New bile duct cannulation technique using a fine-needle aspiration needle to prevent post-endoscopic retrograde cholangiopancreatography pancreatitis

**DOI:** 10.1055/a-2783-4208

**Published:** 2026-02-26

**Authors:** Ikuhiro Kobori, Ou Takagi, Haruka Kato, Masaru Kuwada, Koichi Soga, Yasumi Katayama, Masaya Tamano

**Affiliations:** 1 26263 Department of Gastroenterology, Dokkyo Medical University Saitama Medical Center, Koshigaya, Japan

Endoscopic retrograde cholangiopancreatography (ERCP) carries a risk of post-ERCP pancreatitis (PEP 1 ). Precut techniques and needle-knife fistulotomy carry risks such as perforation and bleeding 2 3 4 . We report a novel method for bile duct cannulation using a fine-needle aspiration (FNA) needle.

**Fig. 1:**
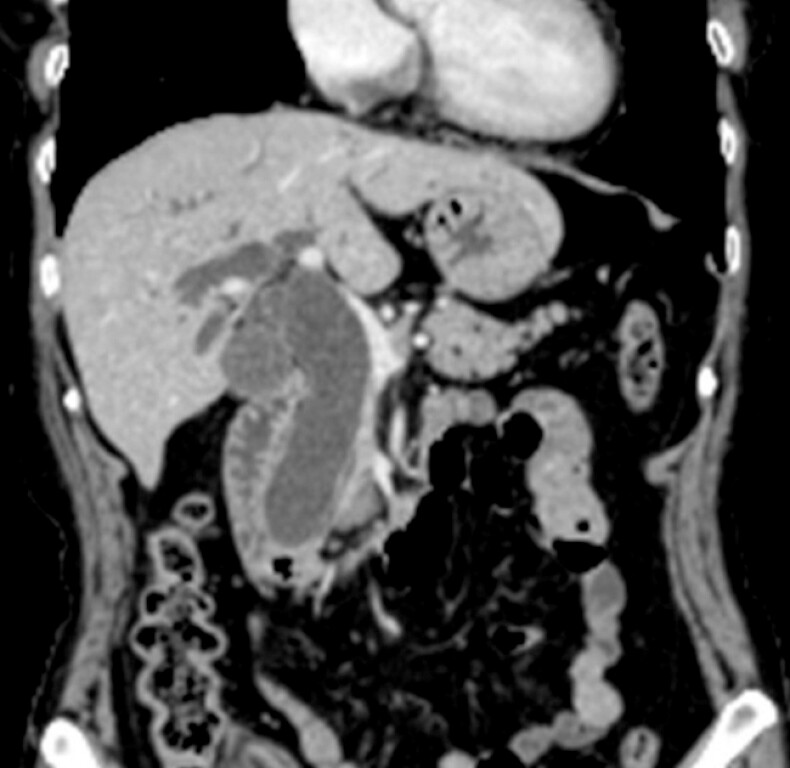
Contrast-enhanced computed tomography shows duodenal papillary carcinoma and dilation of the common bile duct.

**Fig. 2:**
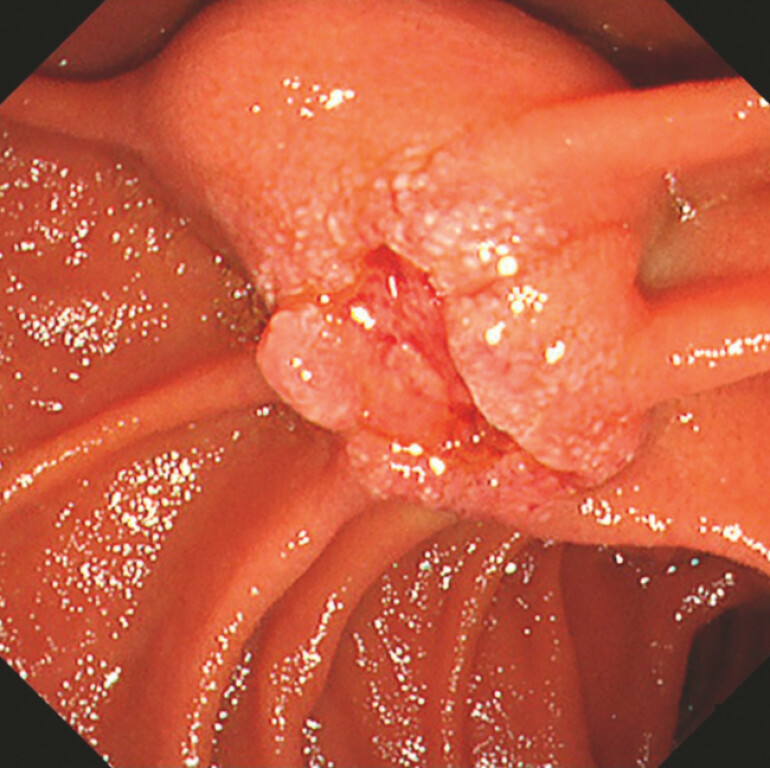
An endoscopic view showing a large oral protrusion and a depressed lesion at the duodenal papilla, with indistinct bile duct openings.

**Fig. 3:**
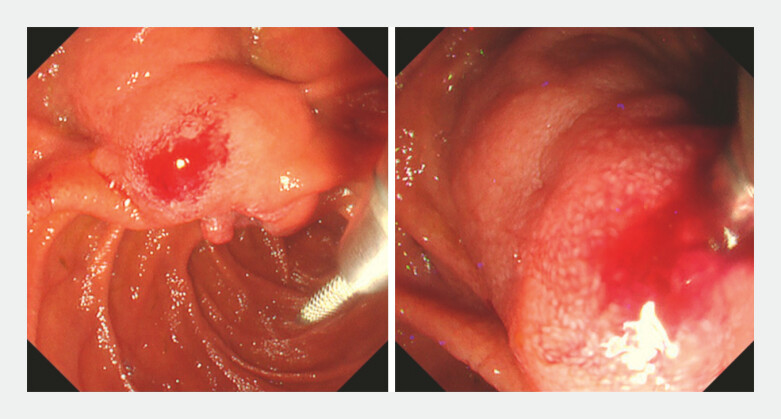
An Endoscopic view showing the puncture of the oral protrusion (left) and insertion of a guidewire into the bile duct through a 22-gauge fine-needle aspiration needle (right).

**Fig. 4:**
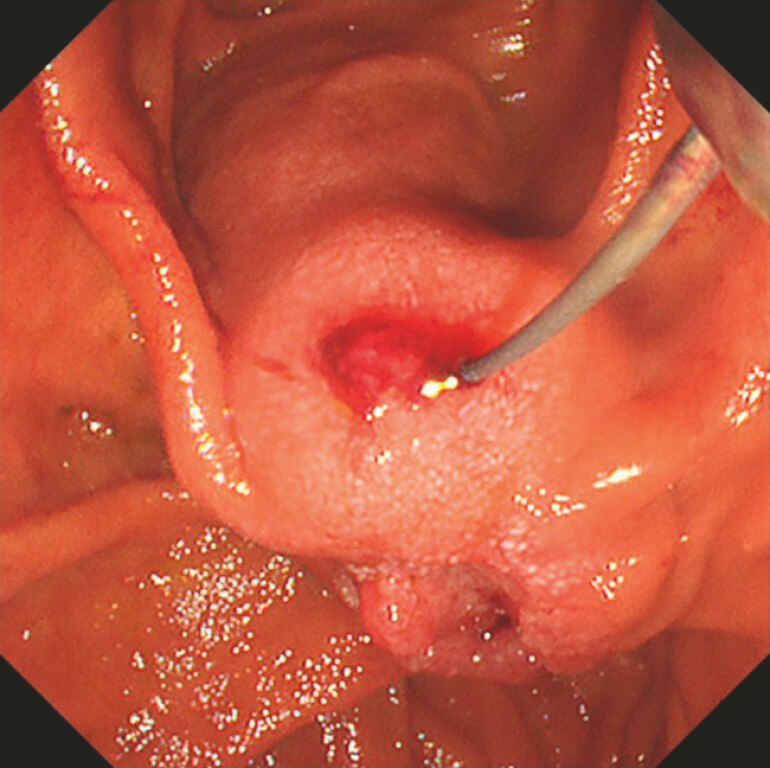
An endoscopic view showing the insertion of a 0.018-inch guidewire into the bile duct through the puncture site.

A 78-year-old woman underwent ERCP for obstructive jaundice due to duodenal papillary carcinoma ( Fig. 1 ). Endoscopic examination revealed a depressed lesion at the duodenal papilla, with indistinct bile duct openings ( Fig. 2 ). To reduce the risk of PEP and bleeding, we decided to perform biliary drainage using a new method that avoids touching the papillary orifice ( Video 1 ). First, we punctured the large oral protrusion near the papillary orifice, where the bile duct was presumed to be located, using a 22-gauge FNA needle (EZ Shot 3 Plus; Olympus Medical Systems, Tokyo, Japan; Fig. 3 ). A 0.018-inch guidewire (Fielder 18; Olympus Medical Systems, Tokyo, Japan) was placed in the bile duct ( Fig. 4 ). The FNA needle was then removed, and a 3-Fr microcatheter (Daimon ERCP catheter; Hanaco Medical, Saitama, Japan 5 ) was advanced over the guidewire and left in the bile duct. After cholangiography, the guidewire was replaced with a 0.025-inch guidewire. A tapered-tip ERCP catheter was advanced into the bile duct to dilate the puncture site and aspirate bile. Finally, a 7-Fr plastic stent was placed. No complications were observed, and jaundice resolution was favorable.

A novel biliary cannulation technique to reduce the risk of post-endoscopic retrograde cholangiopancreatography pancreatitis, bleeding, and perforation by avoiding contact with the papillary orifice.Video 1

**Fig. 5:**
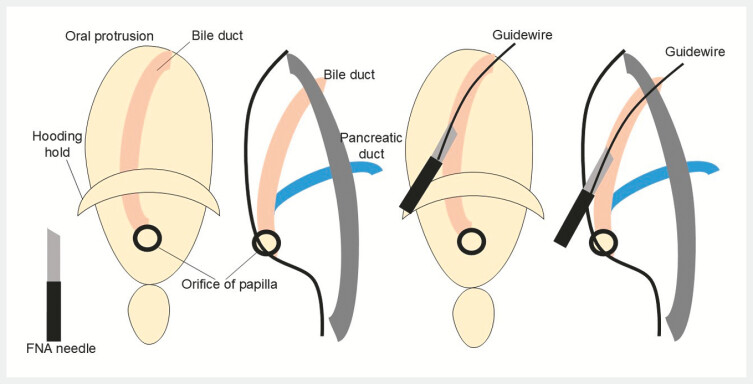
Schema of the puncture of the oral protrusion and insertion of a guidewire into the bile duct through the fine-needle aspiration (FNA) needle.

This method carries an extremely low risk of PEP because it avoids touching the papillary orifice ( Fig. 5 ). It also minimizes the risk of bleeding or perforation by enabling access to the bile duct without applying an electrical current. Even if the bile duct cannot be punctured initially, the small size of the puncture site permits multiple attempts through the adjustment of the puncture location, contributing to the utility of the method.

Endoscopy_UCTN_Code_TTT_1AR_2AC
